# Effects of corn supplementation and age on performance and reproduction of beef females grazing lush spring pasture

**DOI:** 10.1093/tas/txaa046

**Published:** 2020-04-18

**Authors:** Parker A Henley, William T Meteer, Wesley P Chapple, Miles E Redden, Daniel W Shike

**Affiliations:** 1 Department of Animal Sciences, University of Illinois at Urbana-Champaign, Urbana, IL; 2 Orr Research and Demonstration Center, University of Illinois Extension, Baylis, IL

**Keywords:** beef cow, cool-season pasture, excess protein, reproduction, supplementation

## Abstract

This study evaluated how corn supplementation and age of female affected body weight (BW), body condition score (BCS), artificial insemination (AI) pregnancy rate, and blood metabolites (nonesterified fatty acid [NEFA], β-hydroxybutyrate [BHBA], and blood urea nitrogen [BUN]) when grazing lush spring pasture. Angus and Angus × Simmental beef females (*n* = 361) were blocked by location, stratified by BW and BCS, and then were assigned to groups (*n* = 8 groups/treatment combination; 9–14 females/group). The study utilized a stratified, randomized complete block design with a 2 × 2 factorial arrangement of treatments. The four treatment combinations were: yearling heifers receiving no supplement (CON-H); yearling heifers receiving supplement of dry-rolled corn (SUPP-H; 1.81 kg as-fed/heifer/d) for 42 d; 2-yr-old lactating cow-calf pairs receiving no supplement (CON-C); and 2-yr-old lactating cow-calf pairs receiving supplement of dry-rolled corn (SUPP-C; 1.81 kg as-fed/cow/d) for 42 d. Supplementation began at AI (end of April) when cows began grazing tall fescue (*Festuca arundinacea schreb*)-red clover (*Trifolium pratense*) pastures. Pasture forage was collected weekly for analysis. Throughout the study, forage crude protein decreased (*P* < 0.01) over time, but acid detergent fiber (ADF), neutral detergent fiber, dry matter, forage height, and forage mass all increased (*P* < 0.01) over time. Females receiving SUPP tended (*P* = 0.10) to have greater BW and greater BW change over the supplementation period. Supplementation × age effects for BCS were detected (*P* = 0.04); SUPP-H had greater BCS than all other treatment combinations at d 42. Cow BHBA was greater (*P* < 0.01) compared with heifers. Female NEFA increased (*P* < 0.01) from d 12 to 42. Control females had greater (*P* = 0.02) serum NEFA concentrations compared with SUPP females. Control females had greater (*P* = 0.03) BUN concentrations compared with SUPP females. Cow BUN was greater (*P* < 0.01) than heifer BUN. Supplementation effects were not detected (*P* ≥ 0.25) for AI or overall pregnancy rate. In conclusion, there were no supplementation × age interactions excluding d 42 BCS. Supplementation regardless of female age tended to improve d 42 BW and BW change. Cow BHBA and BUN was greater compared with the heifers, whereas the supplemented females had decreased NEFA and BUN. Cows tended to have greater AI pregnancy rates than heifers, but supplementation did not affect AI or overall pregnancy rates.

## INTRODUCTION

Nutrition and reproduction are the two most important factors that drive the financial success of a cow-calf enterprise (Hess et al., 2005). Timely nutritional inputs to the beef female may enhance sustainability by improving reproductive performance of the cowherd (Hess et al., 2005). Most cattle systems in the Midwest calve in early spring between February and April. This timing of calving allows peak lactation and breeding to coincide with lush pasture growth allowing producers to forgo supplementation. However, most forage available to cows during breeding is lush, vegetative grass that is relatively high in protein and moisture, and still may not meet nutritional needs of the cow in this energetically demanding period.

In spring, forage grows quickly, and is very high in moisture, generally <25% dry matter (DM; Doungkamchan et al., 2016). Physical fill with wet forage may limit DM intake (Allen, 1996). Additionally, the lush forage usually contains an increased nitrogen content and minimal carbohydrates. Imbalances of N:Carbohydrate, along with the increased moisture content of the forage can lead to cows entering a negative energy balance (NEB; Arelovich et al., 2003). Supplementing steers grazing lush spring pasture with cottonseed meal improved average daily gain (ADG; Worrell et al., 1990). In dairy cattle, blood urea nitrogen (BUN) levels greater than 20 mg/dL altered uterine environment and decreased the ability of oocytes to develop to blastocysts (Hammon et al., 2005; Santos et al., 2009). In contrast, there was not an improvement in pregnancy rate to artificial insemination (AI) when beef heifers housed in drylot during breeding were compared with heifers grazing wheat pasture although BUN concentrations (5.77 vs. 29.15 mg/dL; respectively) were different (Bryant et al., 2011). Nonetheless, the majority of grazing cow supplementation research has been focused on times when forage quality is the poorest. When mature cows grazing lush spring pasture were supplemented daily, they tended to have decreased BUN levels and, although not statistically significant, numerically greater AI pregnancy rates (51.7% vs. 38.5%; Doungkamchan et al., 2016). Two-yr-old lactating cows represent the greatest reproductive risk in the beef herd, therefore, the percentage of cows that successfully rebreed is dramatically reduced if management steps are not taken to mitigate these effects (Banta et al., 2005). Additionally, transitioning heifers from the drylot to a grazing setting following AI has resulted in reduced reproductive success (Perry et al., 2013) which has been attributed to their lack of grazing experience (Perry et al., 2013) and increased activity level (Perry et al., 2015).

Therefore, the objectives of this experiment were to evaluated how corn supplementation and age of female affected body weight (BW), body condition score (BCS), AI pregnancy rate, and blood metabolites (nonesterified fatty acid [NEFA], β-hydroxybutyrate [BHBA], and BUN) when grazing lush spring pasture. We hypothesized that supplementation of corn daily would improve AI pregnancy rate and overall production performance of beef females. Supplement should dilute the high-nitrogen pasture while offsetting the energetic costs of the heifer activity level and increased nutritional requirements of heifers and 2-yr-old lactating cow, thus preventing a NEB. Additionally, we hypothesized that supplementation would have greatest impact on 2-yr-old lactating cows because of the additional requirements of lactation.

## MATERIALS AND METHODS

All experimental procedures were approved by the Institutional Animal Care and Use Committee of the University of Illinois (IACUC #17292) and followed the guidelines recommended in the Guide for the Care and Use of Agricultural Animal in Agricultural Research and Teaching (FASS, 2010).

### Animals, Experimental Design, and Treatments

Angus and Angus × Simmental beef females (*n* = 361; heifers [*n* = 205; age = 451 ± 23 d; initial BW = 447 ± 41 kg; initial BCS = 5.9 ± 0.62] or cows [cow-calf pairs; *n* = 156; age = 802 ± 27 d; days postpartum = 86 ± 11 d; initial BW = 560 ± 49 kg; initial BCS = 5.5 ± 0.61]) were blocked by location then stratified by BW and BCS and were assigned to groups (*n* = 8 groups/treatment combination; 9–14 females/group) from the end of April through early June in 2017 and 2018. The study utilized a stratified, randomized complete block design with a 2 × 2 factorial arrangement of treatments. The four treatment combinations were: yearling heifers receiving no supplement (CON-H); yearling heifers receiving supplement of dry-rolled corn (SUPP-H; 1.81 kg as-fed/heifer/d) for 42 d; 2-yr-old lactating cow-calf pairs receiving no supplement (CON-C); and 2-yr-old lactating cow-calf pairs receiving supplement of dry-rolled corn (SUPP-C; 1.81 kg as-fed/cow/d) for 42 d. Supplementation began at AI (end of April) when cows began grazing tall fescue (*Festuca arundinacea schreb*)-red clover (*Trifolium pratense*) pastures. Cattle were maintained at two locations: the Beef Cattle and Sheep Field Laboratory in Urbana, IL and the Orr Agricultural Research and Demonstration Center in Baylis, IL.

Prior to the initiation of trial cattle were housed in a drylot and fed a total mixed ration. During this time, cows were limit-fed a corn silage, corn stalks, and modified wet distillers grains diet (11.4 kg of DM/cow/d; 46% neutral detergent fiber [NDF], 25% acid detergent fiber [ADF], 4.0% fat, and 10.2% crude protein [CP]; 1.58 Mcal/kg NEm). Whereas heifers were limit-fed a diet consisting of alfalfa haylage, corn silage, modified wet distillers grains, and supplement (8.2 kg of DM/heifer/d; 41.1% NDF, 21% ADF, 4.2% fat, and 10.5% CP; 1.65 Mcal/kg NEm). Supplementation began at the time of AI (end of April). As typical in a spring-calving herd, breeding often coincides with turnout to spring pastures. Females grazed pastures with an average coverage area of 30% red clover (*Trifolium pratense*) and 70% endophyte-infected fescue (*Festuca arundinacea*) in each pasture. Cattle continuously grazed a pasture for the duration of the experiment. At the Orr Agricultural Research and Demonstration Center in Baylis, IL and the Beef Cattle and Sheep Field Laboratory in Urbana, IL average pasture size was 2.6 ± 0.2 and 3.2 ± 0.5 ha, respectively. This resulted in an average stocking rate of 3.8 cow-calf pairs/ha and 5.0 heifers/ha at the Orr Agricultural Research and Demonstration Center in Baylis, IL and 4.0 cow-calf pairs/ha and 3.8 heifer/ha at the Beef Cattle and Sheep Field Laboratory in Urbana, IL.

Forage mass and nutritive quality were determined by clipping 0.25 m^2^ quadrats (three in each pasture) before the beginning of grazing and every 7 d during the experiment. A 0.25 m^2^ ring was tossed three times while traveling in a zigzag pattern across the pasture to randomly identify the sampling quadrats. Forage within quadrats was mechanically clipped to an above ground stubble height of approximately 7.62 cm. Fresh-cut forage was then placed into a paper bag, weighed then oven dried at 55°C for a minimum of 3 d, and weighed again. Forage mass was calculated for each pasture based on dry-weight data multiplied by the area of the pasture. Forage samples were ground through a 1 mm screen using a Wiley mill (Arthur H. Thomas, Philadelphia, PA) then composited by pasture at each time point. Ground forage was analyzed for NDF and ADF using an Ankom 200 Fiber Analyzer (Ankom Technology, Macedon, NY) as well as CP (Leco TruMac, LECO Corporation, St. Joseph, MI). A commercial mineral (#CP63, Pike Feeds, Pittsfield, IL; 12% to 14% Ca, 8% K, 18% to 20% NaCl, 11% Mg, 90 mg/kg of I, 528,000 IU/kg of vitamin A, 88,000 IU/kg of vitamin D_3_, and 2,200 IU/kg of vitamin E) was offered free choice throughout the experiment.

At the end of the supplementation period, cows were commingled by age within location and grazed common pasture (red clover, white clover, and endophyte-infected fescue). Full BW measurements were collected and averaged at the beginning (d 3 and 0) and end of the experiment (d 41 and 42). Cow BCS were determined at d 3 and d 42 of supplementation and were evaluated using a 1–9 scale (emaciated =1; obese = 9; as described by Wagner et al. [1988]).

### Estrus Synchronization and AI

Cows were synchronized using a 7-d CO-Synch + controlled internal drug-release (CIDR; Pfizer Animal Health, New York, NY) protocol (Larson et al., 2006) and were artificially inseminated at a fixed time. In year 1, heifers were enrolled in a 7-d CO-Synch + CIDR protocol (Lamb et al., 2006) and were artificially inseminated at a fixed time. In year 2, heifers were enrolled in a 14-d CIDR-PG protocol (Mallory et al., 2010) and were artificially inseminated at a fixed time. Sire (*n* = 4) and AI technician (*n* = 4) were stratified across treatment combinations.

In year 1, one bull was added to each pasture group 12 d after AI and remained there for 35 d until conclusion of supplementation period. In year 2, on d 12, heat detection patches (Estrotect Heat Detectors, Rockway Inc., Spring Valley, WI) were placed on all heifers. Trained farm personnel detected estrus and AI females exhibiting estrus 12 h after standing estrus. On d 27, one bull was added to each pasture group. In both years, cows were commingled by age at the end of the supplementation period and one bull was added to each group for 10 d ± 3.5 in both years. Thus, females were exposed to bulls for 43 d in year 1 and 22 d in year 2. Pregnancy diagnoses to AI was performed 42 d after AI in year 1 and 60 d after AI in year 2. Overall pregnancy diagnoses was performed 62 d after bull removal in both years. Pregnancy was confirmed by a trained technician via ultrasonography (Aloka 500 instrument, Wallingford, CT; 7.5-MHz general purpose transducer array).

### Blood Sampling and Analysis

Serum samples collected on d 0, 12, and 42 of supplementation were pooled by pasture group and analyzed for BUN, NEFA, and BHBA concentrations to indicate excess protein intake and reduced energy status. Blood was collected using a 38-mm needle into a 10-mL Vacutainer (Becton, Dickinson and Co., Franklin Lakes, NJ) and stored on ice immediately. Blood in the serum tube was allowed to clot at room temperature before being centrifuged (Model HN-S, International Equipment Company, Needham Heights, MA) at 1,300 × *g* for 20 min at 5°C. About 200 μl from each animal within a group was pooled. Pooled serum was immediately frozen at −20°C until analysis. Composited serum samples were delivered to the University of Illinois, College of Veterinary Medicine Diagnostic Laboratory. Serum BHBA, NEFA, and BUN concentrations were measured in duplicate and analyzed using an Olympus AU680 Chemistry-Immuno Analyzer (Olympus Corporation, Center Valley, PA). Analysis of NEFA was conducted via a colorimetric assay (HR Series NEFA-HR (2); Wako Chemicals, Richmond, VA). Colorimetric analyses were also used for BUN (OSR6134; Beckman Coulter, Indianapolis, IN) and BHBA concentration determination (Ranbut; Randox, Crumlin, UK). The intra- and inter-assay CV were, respectively, 0.8 and 4.9 for NEFA, 2.4 and 2.5 for BUN, and 3.8 and 5.1 for BHBA.

### Statistical Analysis

A stratified, randomized complete block design with a 2 × 2 factorial arrangement of treatments was used and group served as the experimental unit. Pasture forage analysis and pooled blood metabolite data were analyzed using the MIXED procedure of SAS 9.4 (SAS Institute Inc., Cary, NC). The model included the fixed effect of supplementation, age, time, supplementation × age, supplementation × time, age × time, and supplementation × age × time. Baseline values for BHBA, NEFA, and BUN, which were collected at the start of the study, were included as a covariate. Location was included as a block and year was included as a random effect. The REPEATED statement of SAS 9.4 (SAS Institute Inc., Cary, NC) was used to model the repeated measurements within pasture group, and the autoregressive covariance structure was selected for pasture forage quality and the Toeplitz was selected for pooled blood metabolite data after considering the Akaike and Bayesian information criteria.

BW and BCS were also analyzed using the MIXED procedure of SAS 9.4 (SAS Institute Inc., Cary, NC) with the fixed effects of supplementation, age, and the interaction of supplementation and age. Year and group (supplementation × age) was included as a random effect.

Binomial data for AI and overall pregnancy data were analyzed using the GLIMMIX procedure of SAS with the fixed effects of supplementation, age, and the interaction of supplementation and age. Year and group (supplementation × age) was included as a random effect. Technician and AI sire were not significant for AI pregnancy rates and thus were removed from the model. Main effects were considered significant at *P* ≤ 0.05 and tendencies were noted at 0.05 < *P* ≤ 0.10. Means reported in tables are least squares means ± SEM.

## RESULTS

**Figure 1.**
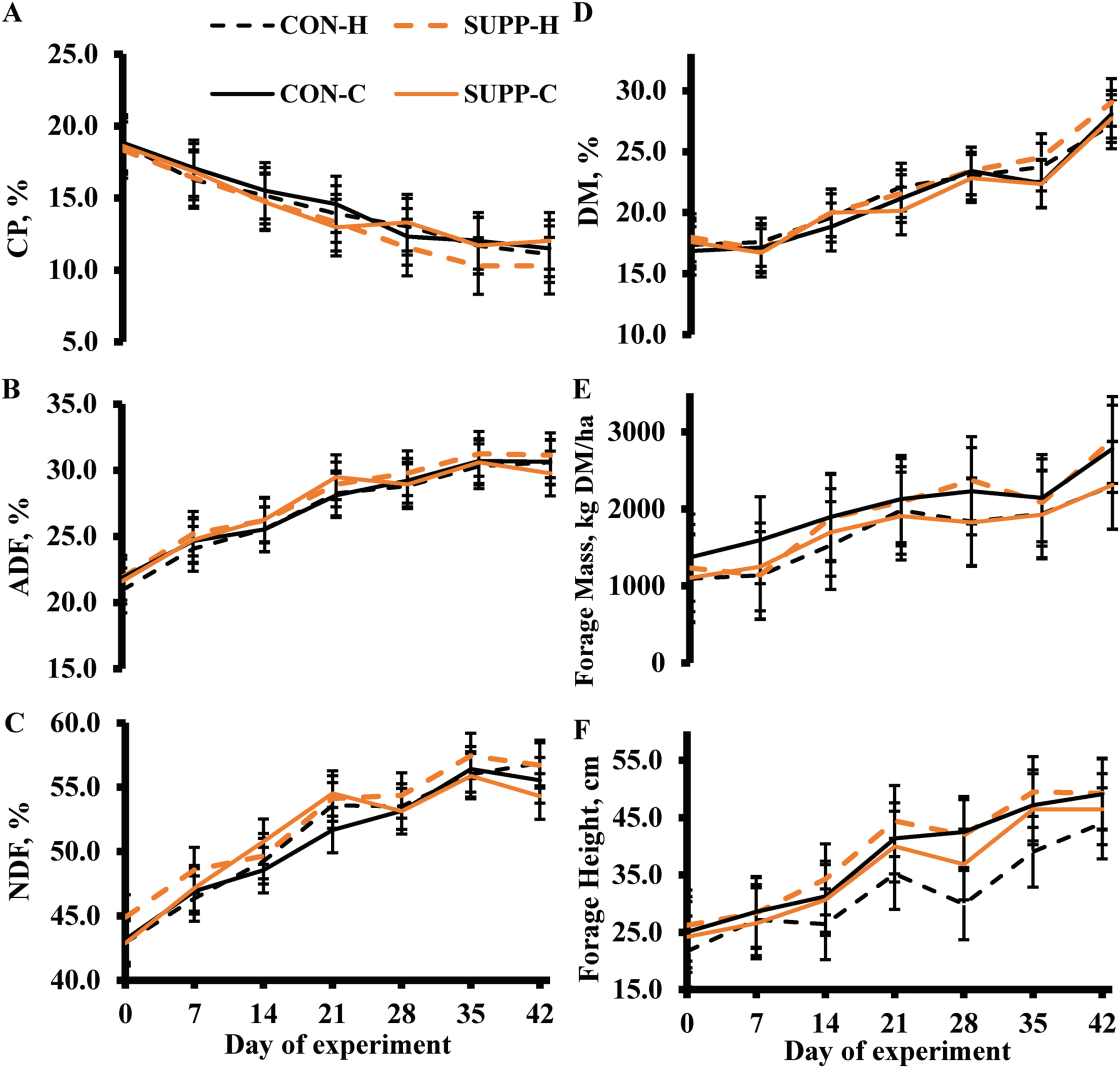
Pasture forage analysis (CP, ADF, NDF, DM, forage mass, and forage height) of the four treatment combinations (*n* = 8 groups per treatment combination): yearling heifers receiving no supplement (CON-H); yearling heifers receiving supplement of dry-rolled corn (SUPP-H; 1.81 kg as-fed/heifer/d) for 42 d; 2-yr-old lactating cow-calf pairs receiving no supplement (CON-C); and 2-yr-old lactating cow-calf pairs receiving supplement of dry-rolled corn (SUPP-C; 1.81 kg as-fed/cow/d) for 42 d from the end of April through early June in 2017 and 2018. Supplementation × age × time, supplementation × age, supplementation × time, age × time, supplementation, and age effects were not detected (*P* ≥ 0.28) for CP, ADF, NDF, or DM. Supplementation × age × time, supplementation × time, age × time, supplementation, and age effects were not detected (*P* ≥ 0.31) for forage height or forage mass. However, a supplementation × age effect was detected (*P* < 0.01) for forage mass and a trend (*P* = 0.08) was detected for forage height. There was a significant time effect (*P* < 0.01) on all forage classification parameters. Error bars = SEM.

Supplementation × age × time, supplementation × age, supplementation × time, age × time, supplementation, and age effects were not detected (*P* ≥ 0.28; Figure 1) for pasture CP, ADF, NDF, or DM percentage. Supplementation × age × time, supplementation × time, age × time, supplementation, and age effects were not detected (*P* ≥ 0.31) for forage height or forage mass. However, a supplementation × age effect was detected (*P* < 0.01) for forage mass and a trend (*P* = 0.08) was detected for forage height. Greater forage heights were noted for SUPP-H compared with CON-H (39.1 vs. 32.0 cm, respectively), yet, the CON-C and SUPP-C were intermediate and were not different from any treatment combination. Furthermore, SUPP-H were in pastures with greater forage mass compared with CON-H (1955 vs. 1686 kg of DM/ha, respectively). Whereas the CON-C had greater forage mass than SUPP-C (2019 vs. 1715 kg of DM/ha, respectively). There was a time effect (*P* < 0.01) on all forage parameters. Forage CP percentage decreased over time, but ADF, NDF, DM percentage, forage height, and forage mass all increased over time.

Supplementation × age effects were not detected (*P* ≥ 0.77; Table 1) for d 0 BW, d 42 BW, or BW change. Age effects were detected (*P* < 0.01) for d 0 BW and d 42 BW, the cows were heavier compared with the heifers. Finally, females receiving SUPP tended (*P* = 0.10) to have greater BW at d 42 and greater BW gain over the supplementation period.

**Table 1.** The effect of corn supplementation on female BW and BCS

	Treatment^*a*^							
	Heifers		Cows			*P*-value^*b*^		
Item	Control	SUPP	Control	SUPP	SEM	Sup	Age	S × A
BW, kg								
d 0	447	447	559	559	5.1	0.99	<0.01	0.98
d 42	447	456	548	554	7.8	0.10	<0.01	0.77
Change	−1	9	−9	0	5.5	0.10	0.12	0.96
BCS								
d 0	5.9	5.9	5.5	5.5	0.20	0.97	<0.01	0.83
d 42	5.5^b^	5.7^a^	5.6^b^	5.6^b^	0.12	0.09	0.27	0.04
Change	−0.4	−0.2	0.1	0.1	0.09	0.32	<0.01	0.38

^*a*^Control= no supplement; SUPP = 1.81 kg as-fed/female/d of dry rolled corn; Heifers = yearling heifers; cows = 2-yr-old lactating cow-calf pairs; *n* = 8 groups per treatment combination.

^*b*^Abbreviations are defined as supplementation effect (Sup) and Supplementation × Age effect (S × A).

Supplementation × age and supplementation effects were not detected (*P* ≥ 0.32) for d 0 BCS and BCS change. However, heifers had a greater d 0 BCS and lost more BCS compared with the cows. Supplementation × age effects were detected (*P* = 0.04) for d 42 BCS and females receiving SUPP tended (*P* = 0.09) for d 42 BCS. Greater BCS was observed for SUPP-H compared with all other treatment combinations, which were not different from each other. Age effects were not detected (*P* = 0.27) for d 42 BCS.

**Figure 2.**
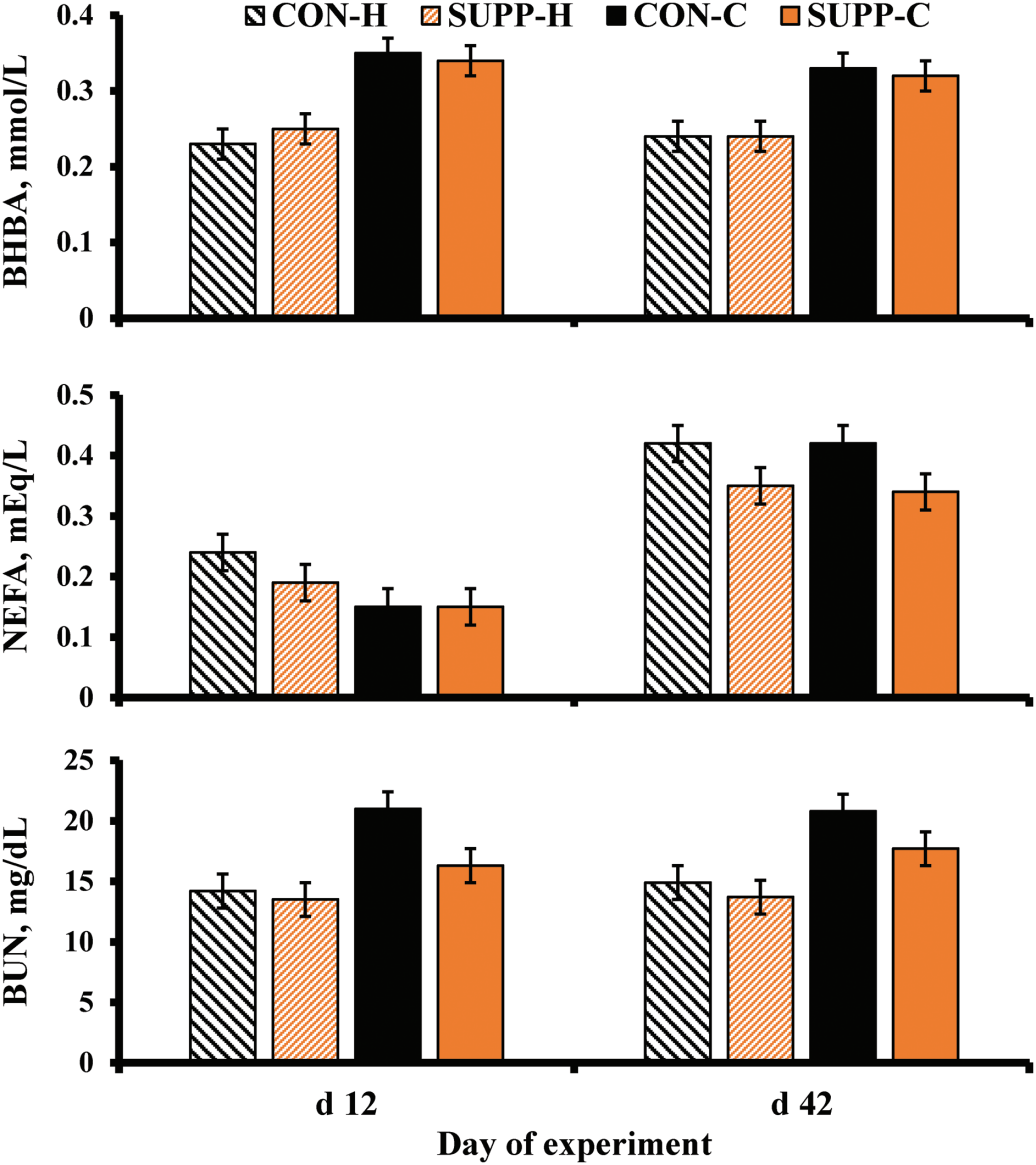
Serum concentration of NEFA, BHBA, and BUN on d 0, 12, and 42 of the four treatment combinations (*n* = 8 groups per treatment combination): yearling heifers receiving no supplement (CON-H); yearling heifers receiving supplement of dry-rolled corn (SUPP-H; 1.81 kg as-fed/heifer/d) for 42 d; 2-yr-old lactating cow-calf pairs receiving no supplement (CON-C); and 2-yr-old lactating cow-calf pairs receiving supplement of dry-rolled corn (SUPP-C; 1.81 kg as-fed/cow/d) for 42 d from the end of April through early June in 2017 and 2018. Supplementation × age × time, supplementation × age, supplementation × time, age × time, supplementation and time effects were not detected (*P* ≥ 0.22) for serum BHBA concentration; but, an age effect was detected (*P* < 0.01). Supplementation × age × time, supplementation × age, supplementation × time, age × time and age effects were not detected (*P* ≥ 0.11) for serum NEFA concentration; however, supplementation and time effects were detected (*P* ≤ 0.02). Supplementation × age × time, supplementation × age, supplementation × time, age × time and time effects were not detected (*P* ≥ 0.18) for serum BUN concentration; but, supplementation and age effects were detected (*P* ≤ 0.03). Error bars = SEM.

Supplementation × age × time, supplementation × age, supplementation × time, age × time, supplementation and time effects were not detected (*P* ≥ 0.22; Figure 2) for serum BHBA concentration; but, an age effect was detected (*P* < 0.01). Cow serum BHBA was greater than heifer BHBA. Supplementation × age × time, supplementation × age, supplementation × time, age × time and age effects were not detected (*P* ≥ 0.11) for serum NEFA concentration; however, supplementation and time effects were detected (*P* ≤ 0.02). Control females had greater serum NEFA concentrations compared with SUPP females. Serum NEFA concentrations were lesser at d 12 than d 42. Finally, supplementation × age × time, supplementation × age, supplementation × time, age × time and time effects were not detected (*P* ≥ 0.18) for serum BUN concentration; but, supplementation and age effects were detected (*P* ≤ 0.03). Control females had greater serum BUN concentrations compared with SUPP females. Serum BUN from the cows was greater than serum BUN from the heifers.

**Figure 3.**
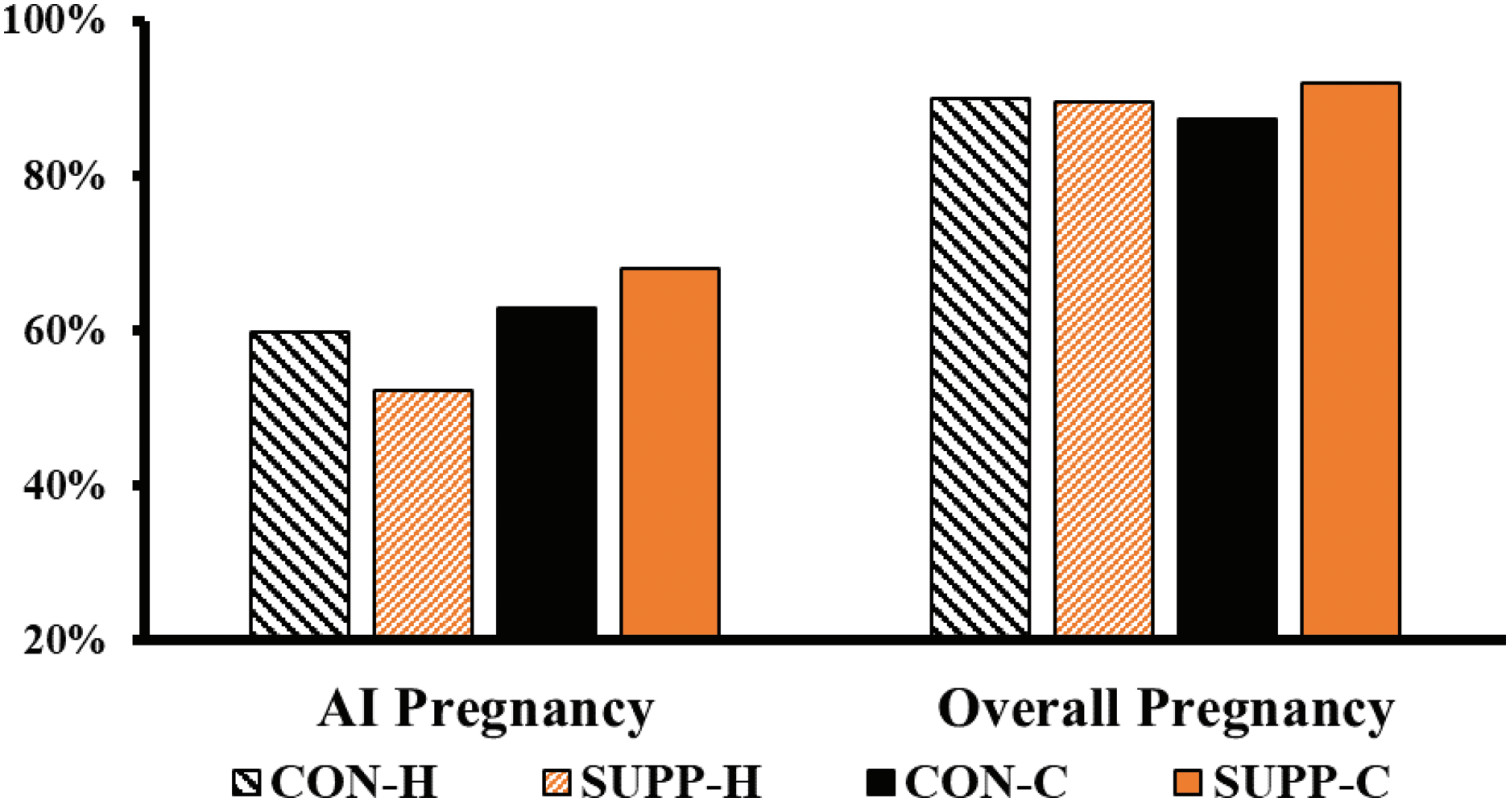
Reproductive performance (AI pregnancy rate and overall pregnancy rate) of four treatment combinations (*n* = 8 groups per treatment combination): yearling heifers receiving no supplement (CON-H); yearling heifers receiving supplement of dry-rolled corn (SUPP-H; 1.81 kg as-fed/heifer/d) for 42 d; 2-yr-old lactating cow-calf pairs receiving no supplement (CON-C); and 2-yr-old lactating cow-calf pairs receiving supplement of dry-rolled corn (SUPP-C; 1.81 kg as-fed/cow/d) for 42 d from the end of April through early June in 2017 and 2018. Supplementation × age and supplementation effects were not detected (*P* ≥ 0.25) for AI or overall pregnancy rate percentage. Age tended (*P* = 0.07) to affect AI pregnancy rate, but not affect (*P* = 0.98) overall pregnancy rates.

Supplementation × age and supplementation effects were not detected (*P* ≥ 0.25; Figure 3) for AI or overall pregnancy rate percentage. However, cows tended (*P* = 0.07) to have a greater AI pregnancy rate compared with the heifers (66% vs. 56%, respectively). Nevertheless, age did not affect (*P* = 0.98) overall pregnancy rates.

## DISCUSSION

Forage CP was equal to or greater than 18% at the initiation of the trial (late April) and remained greater than 10% throughout the entire supplementation period. This is comparable with a similar study by Doungkamchan et al. (2016) that was conducted at the Orr Agricultural Research and Demonstration Center in Baylis, IL, where late-April CP levels were approximately 20% and declined to approximately 13% by early June. Furthermore, early vegetative late-March fescue sampled in Kentucky, averaged approximately 22% CP (Steen et al., 1979) and 24% CP (Beck et al., 2008). Forage CP in the present study declined to approximately 11% by d 35 of supplementation. This is in contrast to previous work, which has noted forage CP only dropping to 13% (Steen et al., 1979; Beck et al., 2008; Doungkamchan et al., 2016) when sampled later in the spring.

Average NDF and ADF of the pastures at d 0 were approximately 43% and 21%, respectively. These results are comparable with the previously mentioned studies by Doungkamchan et al. (2016) and Steen et al. (1979), who found average NDF to be approximately 50% and 43%, respectively, and ADF to be approximately 25% and 30%, respectively.

These CP, NDF, and ADF results indicate that the pastures used in this experiment were vegetative when supplementation began in late April. As the spring commenced, NDF and ADF increased and CP decreased, which is indicative of forage maturity. This experiment’s objective was to challenge the cows with high CP, lush pasture at the time of AI, and it was achieved. The CP content for the first 10 d of supplementation would have been classified as a diet high in CP as defined by Butler (2000; 17% to 19%), which has been associated with decreased reproductive performance (Butler, 2000).

In the present study, DM percentage was equal to or lesser than 20% for the first 14 d of supplementation, then gradually increased to approximately 28% on d 42 (early June). This is similar to the study by Doungkamchan et al. (2016) where DM percentage dropped to 18% in late April and increased to 24% over the following 30 d.

In the present study, forage mass was approximately 1200 kg DM/ha at d 0 and increased to 2500 kg DM/ha over the 42 d supplementation period. Forage height follows a similar trend; at d 0 forage height was 24 cm and increased to 47 cm by d 42. This rapid forage growth facilitates the assumption that forage availability was not limiting. When forage is of higher quality than is required by the grazing animal, forage intake will be depressed when a concentrate feed is given (Kartchner, 1980). This likely explains why the SUPP-H had a greater forage mass and forage height compared with CON-H. However, the fact that the control cows had a greater forage mass is not supported by literature and cannot be explained. Although there were small differences in forage height and forage mass between the treatments, forage availability was not likely limiting.

BW and BCS of the heifers and cows (447 and 559 kg; 5.9 and 5.5, respectively) are different because of their age. We hypothesized that abruptly transitioning young females to lush spring pasture would challenge them. Thus, we expected heifers and cows would lose or maintain BW and BCS over the experimental period. Females receiving SUPP did have a greater BW and BCS at d 42. Greater BCS for the SUPP-H on d 42 is supported in the literature. Perry et al. (2015) transitioned heifers to spring pasture immediately following AI and supplemented them with DDGS (2.2 kg as-fed/d) and compared them with heifers receiving no supplementation. Supplementation resulted in greater BCS (5.9 vs. 5.8) at the end of the supplementation period. Additionally, steers supplemented with energy while grazing irrigated lush pasture had improved ADG (Lake et al., 1974; Anderson and Dunn, 1982). In contrast, Doungkamchan et al. (2016) observed no differences in final BW or BCS after supplementing mature cows for 62 d with 1.81 kg of DM/cow/d. Differences in BCS between the CON-H and SUPP-H may be attributed to heifers being naive to grazing. Pervious work has noted that heifers developed in a drylot and subsequently turned out to pasture exhibited reduced ADG during the first 27 d (Perry et al., 2013) and increased activity level during the first 3 d compared with their range developed counterparts (Perry et al., 2015). In the current study, the 0.2 BCS difference between SUPP-H and CON-H being statistically different can be attributed to a small standard error and is likely not biologically different.

BHBA is a ketone body used to reflect the completeness of fat oxidation in the liver and when elevated above 1.20 mmol/L, which is the threshold for a healthy cow, can result in poorer milk production and reduced reproductive performance in dairy cattle (LeBlanc et al., 2010). Females in this experiment had serum BHBA concentrations ranging between 0.2 and 0.4 mmol/L, which indicates that the cows were not ketotic. Thus, the difference in BHBA between heifers and cows is likely not biologically significant.

During periods when digested energy supply is inadequate to meet the cow’s nutritional requirements, such as during lactation, stored lipids are mobilized in the form of NEFA for conversion to energy (Richards et al., 1989). According to Garverick et al. (2013), dairy cows with a NEFA concentration greater than 0.20 mEq/L will use more adipose tissue to meet their energy requirement and are more likely to experience BCS loss. Females receiving SUPP had decreased NEFA, which can be attributed to the additional dietary energy being supplied by the corn supplement, which may prevent those females from entering a NEB. Overall, it appears that cows were mobilizing stored energy which resulted in an increased NEFA concentration. But, their BHBA levels were normal, so their livers were able to accommodate the increased fatty acid oxidation.

The difference in BUN concentration between control and supplemented females may be due to the dilution effect of the corn supplement. A similar reduction in BUN was noted when mature cows were supplemented 1.81 kg/cow/d of a 45% ground corncobs, 45% soybean hulls, and 10% dry molasses supplement on a DM basis (Doungkamchan et al., 2016). This dilution effect has also been observed in steers grazing irrigated pasture that were provided an energy supplement (Lake et al., 1974). Control cows had BUN values that exceeded the threshold (20 mg/dL) classified by Hammon et al. (2005) which is noted to result in decreased reproductive performance. Yet, there were no differences in AI pregnancy rate between CON-C and SUPP-C. The BUN level and relationship noted in the cows was not observed in the heifers. Authors hypothesized that young females would be most likely to compromise their reproductive system following a drastic diet alteration such as demonstrated in this experiment. However, these heifers may not have been actually consuming as much of the forage. Grazing is a learned behavior that animals acquire from adults prior to weaning (Perry et al., 2013). Upon heifers leaving a drylot situation and assuming a grazing role heifers have been noted to have reduced ADG and increased activity (Perry et al., 2013, 2015).

There were no differences in AI or overall pregnancy rate percentage except cows tended to have a greater AI pregnancy rate compared with the heifers (66% vs. 56%, respectively). This contradicts our hypothesis that supplementation would alleviate the effects of lush spring pasture forage and improve reproductive performance while having the greatest impact in the 2-yr-old cows. In a study that compared the reproductive performance of beef heifers either housed in a drylot or grazing wheat pasture during the breeding season noted that although BUN concentrations (5.77 vs. 29.15 mg/dL; respectively) were different there were no differences in pregnancy rate (Bryant et al., 2011). Also, Gunn et al. (2014) fed diets differing in protein from late gestation until rebreeding and found differences in BUN, but these differences did not translate to significant differences in pregnancy rate. Similarly, in our experiment, BUN concentration of CON-C at d 12 was above the threshold associated with decreased fertility (20 mg/dL) but did not result in a reduction in AI pregnancy rate. This was not expected because high BUN concentrations alter uterine pH and environment (Rhoads et al., 2004). If the uterine environment is in this altered state 10–17 d following ovulation, long-term embryo survival may be compromised (Rhoads et al., 2004). However, a BUN beyond the threshold does not always result in significant reproductive performance differences as Moriel et al. (2012) found. Heifers housed in the drylot and fed low or medium quality hay had had no difference in AI conception rate when BUN concentrations were 17.2 and 22.8 mg/ dL, respectively (Moriel et al., 2012). Other work by Rhoads et al. (2006) investigated the quality of oocytes flushed from superovulated lactating cows having moderate or high BUN concentrations. Dairy cows with BUN concentrations > 19 mg/dL produced oocytes that had decreased viability. Authors attributed the decrease to toxic effects of urea on the oocyte or the early embryo. In the present study, cows where not exposed to the high CP forage until following AI. The reproductive performance issues attributed to elevated BUN are likely multifactorial, by not exposing cows to high CP diets during oocyte development may have prevented the effects that elevated nitrogen levels have on AI pregnancy rate.

In conclusion, there were no supplementation × age interactions excluding d 42 BCS. Supplementation regardless of female age tended to improve d 42 BW and BW change. Cow BHBA and BUN was greater compared with the heifers whereas the supplemented females had decreased NEFA and BUN. Cows tended to have greater AI pregnancy rates than heifers, but supplementation did not affect AI or overall pregnancy rates.
